# Amygdala Lesions Reduce Anxiety-like Behavior in a Human Benzodiazepine-Sensitive Approach–Avoidance Conflict Test

**DOI:** 10.1016/j.biopsych.2017.01.018

**Published:** 2017-10-01

**Authors:** Christoph W. Korn, Johanna Vunder, Júlia Miró, Lluís Fuentemilla, Rene Hurlemann, Dominik R. Bach

**Affiliations:** a Division of Clinical Psychiatry Research, Psychiatric Hospital, Zurich, Switzerland; b Neuroscience Center Zurich, University of Zurich, Zurich, Switzerland; c Epilepsy Unit, University Hospital of Bellvitge, Barcelona, Spain; d Cognition and Brain Plasticity Unit, Institute of Biomedicine Research of Bellvitge, Barcelona, Spain; e Department of Cognition, Development, and Educational Psychology, Barcelona, Spain; f Institute of Neurosciences, University of Barcelona, Barcelona, Spain; g Department of Psychiatry and Division of Medical Psychology, University of Bonn, Bonn, Germany; h Wellcome Trust Centre for Neuroimaging, University College London, London, United Kingdom

**Keywords:** Anxiety disorder, Double-blind, Hippocampus, Lorazepam, Placebo-controlled, Urbach-Wiethe syndrome

## Abstract

**Background:**

Rodent approach–avoidance conflict tests are common preclinical models of human anxiety disorder. Their translational validity mainly rests on the observation that anxiolytic drugs reduce rodent anxiety-like behavior. Here, we capitalized on a recently developed approach–avoidance conflict computer game to investigate the impact of benzodiazepines and of amygdala lesions on putative human anxiety-like behavior. In successive epochs of this game, participants collect monetary tokens on a spatial grid while under threat of virtual predation.

**Methods:**

In a preregistered, randomized, double-blind, placebo-controlled trial, we tested the effect of a single dose (1 mg) of lorazepam (*n* = 59). We then compared 2 patients with bilateral amygdala lesions due to Urbach-Wiethe syndrome with age- and gender-matched control participants (*n* = 17). Based on a previous report, the primary outcome measure was the effect of intra-epoch time (i.e., an adaptation to increasing potential loss) on presence in the safe quadrant of the spatial grid. We hypothesized reduced loss adaptation in this measure under lorazepam and in patients with amygdala lesions.

**Results:**

Lorazepam and amygdala lesions reduced loss adaptation in the primary outcome measure. We found similar results in several secondary outcome measures. The relative reduction of anxiety-like behavior in patients with amygdala lesions was qualitatively and quantitatively indistinguishable from an impact of anterior hippocampus lesions found in a previous report.

**Conclusions:**

Our results establish the translational validity of human approach–avoidance conflict tests in terms of anxiolytic drug action. We identified the amygdala, in addition to the hippocampus, as a critical structure in human anxiety-like behavior.

Preclinical rodent models of anxiety disorders commonly involve a conflict between approach and avoidance motivation (1, 2, 3). This conflict can arise from the drive to explore versus the adversity of unprotected exploration as exemplified in the elevated plus maze (4, 5), open field test (6), and light–dark box (7). In other often-used paradigms, a tendency to approach rewards (e.g., food or water) conflicts with avoidance of negative consequences as in Geller-Seifter and Vogel operant conflict tests involving electric shocks (8, 9) and in novelty-suppressed feeding tests (10). In all of these approach–avoidance conflict (AAC) paradigms, acute administration of benzodiazepines and other anxiolytics reliably reduces adaptation to threat; i.e., animals behave less cautiously (1, 2, 3). The same substances also relieve clinical manifestations of anxiety in humans (1, 2, 3); [see (11, 12) for reviews of pharmacotherapy in clinical anxiety]. This pharmacological evidence constitutes the main argument for the translational validity of AAC paradigms as models of human anxiety disorders. Still, the cross-species validity of AAC is not firmly established given that suitable preclinical test beds for humans have only recently been developed in the form of computer games (13, 14, 15, 16, 17, 18). The neural implementation of human anxiety-like behavior thus remains elusive.

A plethora of studies have addressed the neurobiological implementation of rodent anxiety-like behaviors. They have consistently shown that hippocampal theta oscillations are increased during AAC (19) and that ventral hippocampus lesions have effects similar to anxiolytics (20, 21, 22). However, rodent amygdala lesions did not affect innate anxiety-like behaviors and rendered behavior in AAC with overt rewards more cautious rather than less cautious (23). This is in contradistinction to a role of the amygdala not only in eliciting acute fear responses but also in modulating anxiety-like behavior to context (24, 25, 26).

Interestingly, the amygdala is rich in the molecular targets of benzodiazepines (27), namely gamma-aminobutyric acid A (GABA_A_) receptors (28, 29). In rodents, local administration of benzodiazepines into the amygdala has anxiolytic effects in operant conflict tests and the light–dark box (27). In humans, the amygdala is critically required for storing threat memories (30), and benzodiazepine administration reduced amygdala activity in one human neuroimaging study (31) [see also (32)]. Thus, it appears plausible that benzodiazepines may reduce anxiety-related behavior by inhibiting amygdala neurons in addition to potential effects in the hippocampus. While some functional neuroimaging studies on human AAC have reported involvement of the hippocampus (15, 16), others report activity of the amygdala (13, 14). Results from a lesion study suggest causal involvement of the human hippocampus in AAC behavior (15).

Here, we sought to investigate the impact of benzodiazepines and of amygdala lesions on human AAC. In our behavioral task (15) (Figure 1), participants forage for monetary tokens in successive epochs under threat of a virtual predator that can take away these tokens. Thus, this task explicitly pits rewards (monetary tokens) against punishment (capture by predator and thus loss of previously collected tokens).

**Figure 1:**
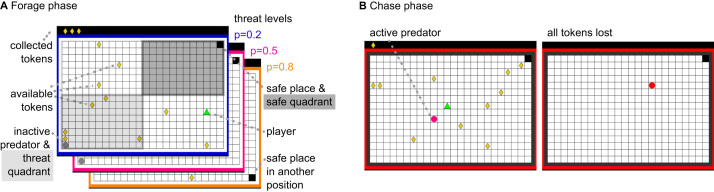
Behavioral approach–avoidance paradigm. **(A)** The human player (green triangle) is foraging for tokens (yellow rhombi), which contribute to financial reimbursement at the end of the game. At any time, 10 tokens are present and are replaced in random position when collected. Collected tokens are shown in the upper left corner, above the grid. Meanwhile, a predator (gray circle) is inactive in a corner of the grid and can attack the human player at any given time according to three probabilities specified by the frame color. To avoid being caught by the predator, the player can seek shelter in a safe place. Presence in the safe quadrant, which surrounds the safe place, constitutes the primary outcome measure. The safe place and thus the safe quadrant are always diagonal to the initial predator position and the surrounding threat quadrant (positions are randomized across epochs). In active epochs, the player and the predator start in the same corner. In passive epochs, the human starts in the safe place. **(B)** When the predator becomes active and starts to chase the player, the frame color turns red. All tokens from this epoch are lost when the player is caught.

Normatively, an agent should adapt behavior over time within an epoch [cf. (17)]. As the number of collected tokens increases over time, potential loss increases and participants should become more cautious by staying close to the safe place. Based on our previous report (15), we defined linear intra-epoch adaptation of presence in the safe quadrant (i.e., the quarter of the field surrounding the safe place) as the primary outcome measure. In other words, we hypothesized linear drug × time and lesion × time interactions in this measure, with a smaller effect of time under lorazepam and in patients with lesions. As secondary outcomes, we investigated six further behavioral metrics (15). In our previous investigation (15), these metrics were intercorrelated—similar to measures from rodent AAC (33, 34). Therefore, we also introduced a summary score that quantifies loss adaptation over intra-epoch time as a weighted average of the rate of change per time unit across all seven metrics.

We first inquired whether human anxiety-like behavior in AAC is reduced by lorazepam as it is in rodents. Next, we sought to quantify to what extent the amygdala contributes to anxiety-like behavior by comparing 2 patients with relatively specific lesions of the bilateral amygdala due to congenital Urbach-Wiethe syndrome (35, 36, 37, 38) with age- and gender-matched healthy individuals and contrasting these results with a previously reported sample of patients with hippocampus lesions due to sclerosis (15).

## Methods and Materials

### Lorazepam Study: Participants, Study Medication, Ethics, and Registration

Participants were recruited from the general population (overall *n* = 60, 30 per group, 15 female participants per group; one female participant was excluded from analysis due to a suspected medical condition). The age of the resulting sample (mean ± SD) was 25.1 ± 4.4 years and did not differ between groups (*p* > .10).

The study medication was 1 mg of oral lorazepam (Temesta, Pfizer, Zurich, Switzerland). Peak plasma concentrations are reached after approximately 120 minutes (39, 40). See also Supplemental Methods.

The study was conducted in accord with the Declaration of Helsinki and approved by the governmental research ethics committee (Kantonale Ethikkomission Zurich, KEK-ZH-Nr. 2014-0196) and the Swiss Agency for Therapeutic Products (Swissmedic, 2014DR1113). All participants gave written informed consent. The study was preregistered at the Swiss Federal Complementary Database (SNCTP000001227) and at the World Health Organization International Clinical Trials Registry Platform (ISRCTN12590498; http://www.isrctn.com/ISRCTN12590498).

### Amygdala Study

Two female monozygotic twins (age 40 years) with selective bilateral amygdala lesions due to Urbach-Wiethe syndrome were tested at the University of Bonn (Germany). Seventeen healthy female participants served as the control group (age 40.2 ± 3.2 years) and were tested at the University of Zurich (Switzerland). The study was conducted in accord with the Declaration of Helsinki and approved by the respective research ethics committees (Bonn: 037/11; Zurich: KEK-ZH-Nr. 2013-0118). All participants gave written informed consent.

Neurological and psychological examinations of the 2 patients with lesions have been extensively reported (35, 36, 37, 41). High-resolution computer-assisted tomography images showed that the calcified volumes include the entire basolateral amygdala and most other amygdala nuclei [see Figures 1 and S3 in (35)]. The hippocampus itself is free of calcifications. There are mild calcifications at the border region between the amygdala and hippocampus (35).

The two patients did not meet criteria for any psychiatric disorder and were not taking any psychotropic medication at the time of testing. One of the twins suffered a first grand mal seizure at age 12 years but stopped anticonvulsive therapy with a then 900-mg daily dose of valproate in 2006, when she became pregnant (35); the other patient never had seizures. Both patients reported pre-epileptic auras that occur up to twice a month.

An extensive neuropsychological test battery conducted at age of 34 years, reported in (41), showed no signs of anxiety or depression (21-Item Hamilton Depression Rating Scale, Hamilton Anxiety Rating Scale, and Beck Depression Inventory II) and no psychopathological symptoms (Symptom Checklist-90–Revised). Both twins had average intelligence as well as intact verbal learning and memory [as assessed by, among others, the Verbaler Lern- und Merkfähigkeitstest, a German version of the Rey Auditory Verbal Learning Test; see (41, 42)]. Their executive functions were average (as measured with the Trail Making Test, the Wisconsin Card Sorting Test, and a Stroop test), but there were impairments in phonemic fluency and short-term concentration. See Supplemental Methods for information on the patients with hippocampus lesions.

### Behavioral Approach–Avoidance Paradigm

Participants completed 240 epochs of our previously described AAC task on a standard PC computer (15), divided into five blocks with short self-paced breaks. In each epoch, participants could move over a grid of 24 × 16 blocks to collect monetary tokens under the threat of being attacked by a predator, which resulted in the loss of all tokens collected in the given epoch. One corner of the grid was a safe place in which the predator could not attack. We refer to the quarter of the grid in which the safe place was located as the safe quadrant (constituting 12 × 8 grid blocks). The location of the safe place was randomized in each epoch.

#### Tokens

At all times 10 tokens were present on the grid, uniformly distributed in space, and every 2 seconds 1 of the tokens changed its position randomly. When participants collected a token, it was added to a row in the upper left corner of the computer screen (above the grid) and a new token appeared in a different place on the grid.

#### Predator

In the beginning of each epoch, a predator was inactive in a corner of the grid (diagonal to the safe place). The threat quadrant constituted the quarter of the grid in which the inactive predator resided (12 × 8 blocks) and was always diagonal to the safe quadrant. The predator could become active and chase participants any time but could not enter the safe place. The color of the frame around the grid (blue, purple, and orange) indicated three distinct predator wake-up probabilities (0.2, 0.5, and 0.8). These threat probabilities were not explicitly revealed beforehand; participants learned to distinguish the different threat levels during the game. Threat levels were balanced across epochs.

#### Active Versus Passive Start

Participants started each epoch either in the same corner as the predator (active start) or from the safe place (passive start). The starting corner was balanced across epochs. See also Supplemental Methods.

### Statistical Analyses of the Behavioral Paradigm

We analyzed participants’ positions on the grid over 1-second bins. Because epoch duration was variable, more data were available for earlier time bins than for later time bins. Presence in the safe quadrant during the foraging phase constituted our primary outcome measure. We also analyzed the following six secondary outcome measures: 1) distance from the threat (i.e., from the predator), 2) distance from the nearest wall, 3) presence in the safe place, 4) presence in the threat quadrant, 5) token collection, and 6) speed when outside the safe place. Our factorial design included a between-subjects factor (lorazepam/placebo or amygdala lesions/healthy control participants) and three within-subjects factors: task (active/passive start), threat level of predator (low/medium/high), and time (15 time bins of 1-second duration). Secondary outcome measures were Bonferroni corrected to account for multiple comparisons. For ease of presentation, and to facilitate comparison of significance across primary, secondary, and additional measures, we state *p* values multiplied by the number of measures in the correction rather than adapting significance thresholds. Resulting values exceeding 1 are stated as 1.

We used the software package R to perform full multistratum repeated-measures analysis of variance (ANOVA) models with Greenhouse-Geisser corrected degrees of freedom and Bonferroni correction for six measures per experiment for the secondary outcomes. Patients with selective amygdala lesions are extremely rare, and often studies need to rely on single cases (35, 43, 44). Our experimental design necessitated a parametric three-factorial analysis for which no single-case statistics are available, unlike for simpler experimental designs (45). Crucially, using multilevel repeated-measures ANOVA models considerably mitigates the concern of limited sample size because all individual responses enter the design matrix under the assumption of equal variance across all cells of the design.

For each of the seven measures, we computed subject-by-subject regression models for the linear effect of time (i.e., 15 time bins). We weighted the individual measures according to their respective theoretical maximum range and summed them to a loss adaptation score. To validate this score, we show that it was significantly reduced in patients with hippocampal sclerosis (*n* = 7) compared with healthy control participants from our previous data set (15) (*n* = 12; *t*_17_ = 2.8; *p* = .0135, two tailed). In the current data sets (lorazepam and amygdala studies), we report one-tailed tests because we had a directional hypothesis.

## Results

### Lorazepam Reduces Anxiety-like Behavior in a Randomized, Double-Blind, Placebo-Controlled Study

**Figure 2:**
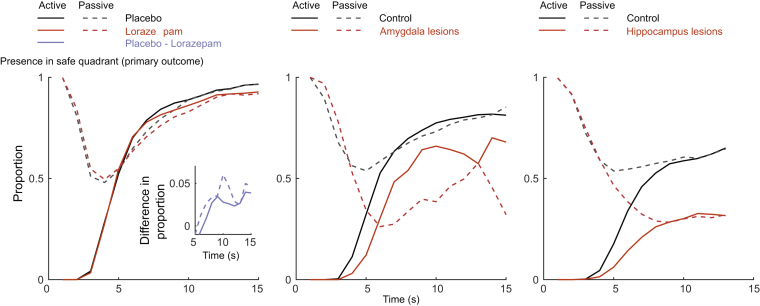
Primary metric of loss adaptation in the approach–avoidance paradigm. The proportion of time spent in the safe quadrant (see Figure 1) shows group differences for lorazepam-treated vs. placebo-treated healthy participants as well as for amygdala and hippocampus lesion patients vs. respectively matched control participants (see Table 1 for statistical results, Figure 3 for results on the six secondary outcome measures, and Figure 4 for results on the loss adaptation score that combines the seven measures). As intra-epoch time progresses, the control groups spent more time in the safe quadrant than the drug and lesion groups (in both active and passive epochs; i.e., the black lines are above the dark red lines for all three experiments). The inset in the left column depicts group differences. As per task design, the initial phase of the epoch is characterized by substantial differences between active epochs (in which the player and the predator start in the same corner) and passive epochs (in which the participant starts in the safe place).

**Figure 3:**
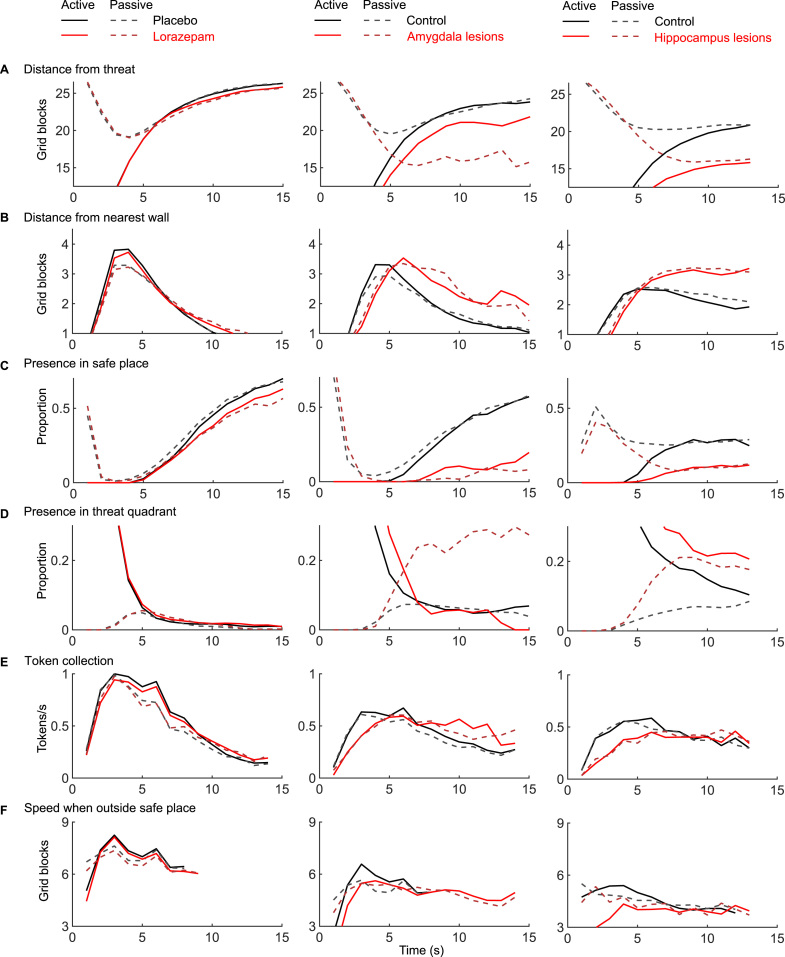
Six secondary metrics of loss adaptation in the approach–avoidance paradigm. Individual metrics show group differences for lorazepam-treated vs. placebo-treated healthy participants as well as for amygdala and hippocampus lesion patients vs. respectively matched controls (see Table 1 for statistical results, Figure 2 for results on the primary outcome measures, and Figure 4 for results on the loss adaptation score that combines the seven measures). In the lorazepam experiment, significant linear interactions of drug × time emerged in four measures (after Bonferroni correction); toward the end of the epoch, participants under lorazepam (compared with those under placebo) were closer to the threatening predator **(A)**, were farther from the nearest wall **(B)**, spent less time in the safe place **(C)**, and collected more tokens **(E)**. Testing patients with amygdala lesions revealed a significant group × time interaction in five measures (after Bonferroni correction); as the epoch progressed, patients with amygdala lesions (compared with controls) were relatively closer to the threatening predator **(A)** and relatively farther from the nearest wall **(B)**, they spent less time in the safe place **(C)**, collected more tokens **(E)**, and were faster when outside the safe place **(F)**. For presence in the threat quadrant **(D)**, no significant linear interactions of group and time were observed in the lorazepam or amygdala study. See Supplemental Figure S3 for graphs depicting the 2 patients separately. Mean data are plotted for time points in which data from all participants are available (e.g., speed outside the safe place is not shown for some later time points because many participants tend to reside in the safe place toward the end of the epoch).

**Figure 4:**
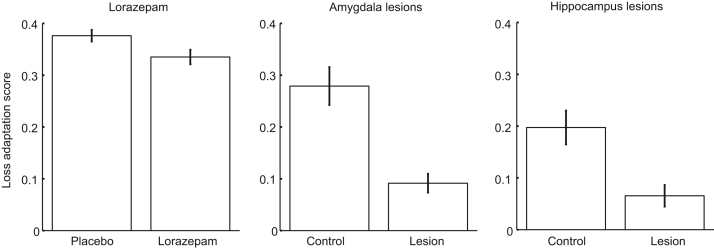
Loss adaptation scores in the approach–avoidance paradigm. Loss adaptation in the game is reduced under lorazepam vs. placebo and in two patients with amygdala lesions vs. healthy control participants. This mirrors reductions of loss adaptation in patients with hippocampus lesions [reanalyzed from (15)]. The loss adaptation score combines the seven behavioral metrics of the approach–avoidance conflict paradigm (see Figures 2 and 3 and Supplemental Figures S1 and S2). The overall differences in loss adaptation scores among the three groups are likely due to age differences (mean ± SD of the three samples in years: lorazepam study: 25.1 ± 4.4; amygdala lesions study: 40.2 ± 3.0; hippocampus lesions study: 44.0 ± 9.3). As a side finding, we observed that loss adaptation score correlated negatively with age across the three control groups (Pearson’s *r* = −.41, *p* = .0013). Error bars depict standard errors of the mean.

Lorazepam had a significant impact on our primary outcome. As intra-epoch time passed, participants under placebo spent increasingly more time in the safe quadrant, and this linear change over time was reduced in participants under lorazepam (*t* = −4.3, *p* < .0001) (Table 1 and Figure 2). In secondary measures, we found a similar linear drug × time interaction in four measures after Bonferroni correction; participants under lorazepam kept less distance from the threat (*t* = −4.2, *p* = .0003) and from the nearest wall (*t* = 5.3, *p* < .0001), had less presence in the safe place (*t* = −5.3, *p* < .0001), and collected more tokens (*t* = 5.3, *p* < .0001) as the epoch progressed compared with those under placebo (Table 1 and Figure 3). This pattern of results was mirrored by a significantly smaller loss adaptation score in the lorazepam group (*t*_57_ = 2.3, *p* = .0134) (Figure 4).

**Table 1:** Primary and Six Secondary Metrics of Loss Adaptation

	Presence in Safe Quadrant (Primary Outcome)		Distance From Threat		Distance From Nearest Wall		Presence in Safe Place		Presence in Threat Quadrant		Token Collection		Speed When Outside Safe Place	
Measure/Effect	*F*/*t* Value	*p* Value	*F*/*t* Value	*p* Value	*F*/*t* Value	*p* Value	*F*/*t* Value	*p* Value	*F*/*t* Value	*p* Value	*F*/*t* Value	*p* Value	*F*/*t* Value	*p* Value
Lorazepam Vs. Placebo Groups														
Group	−0.85	.3987	−1.18	1	0.64	1	−1.33	1	0.67	1	−0.05	1	−1.22	1
Group × threat overall	0.91	.3789	1.09	1	0.30	1	0.27	1	5.55	.0570	0.26	1	0.18	1
Group × threat linear	0.56	.5745	0.88	1	−0.76	1	−0.57	1	−2.11	.2234	−0.34	1	0.27	1
Group × task	−0.19	.8511	−0.52	1	2.24	.1722	−2.20	.1926	−0.07	1	2.29	.1530	−0.45	1
Group × time overall	1.78	.1597	1.63	1	2.69	.3960	2.20	.7524	0.33	1	2.62	.3996	1.36	1
Group × time linear	−4.34^a^	<.0001^a^	−4.16^a^	.0003^a^	5.25^a^	<.0001^a^	−5.34^a^	<.0001^a^	0.09	1	5.34^a^	<.0001^a^	1.29	1
Group × threat × time overall	0.63	.7466	0.83	1	0.70	1	0.80	1	1.19	1	0.75	1	0.49	1
Amygdala Lesions Vs. Healthy Groups														
Group	−1.59	.1305	−1.81	.525	0.83	1	−1.57	.8022	1.77	.5652	0.26	1	−0.72	1
Group × threat overall	0.31	.6901	1.80	1	0.49	1	0.18	1	3.89	.1812	0.47	1	0.54	1
Group × threat linear	0.58	.5660	0.35	1	0.46	1	−0.60	1	−0.82	1	−0.15	1	0.78	1
Group × task	−2.34^a^	.0321^a^	−2.87	.0642	0.39	1	−0.99	1	3.30^a^	.0252^a^	1.37	1	2.31	.204
Group × time overall	2.23	.1311	2.09	.9498	2.37	.6498	2.81	.6096	0.71	1	2.30	.6300	3.21	.1506
Group × time linear	−4.48^a^	.0001^a^	−5.17^a^	.0002^a^	4.14^a^	.0013^a^	−6.20^a^	<.0001^a^	1.91	.3783	4.72^a^	.0002^a^	3.68^a^	.0031^a^
Group × threat × time overall	1.55	.1474	1.43	1	1.20	1	0.31	1	1.86	.2664	1.09	1	1.17	1

Comparison of lorazepam-treated (*n* = 29) and placebo-treated (*n* = 30) participants and comparison of 2 patients with bilateral amygdala lesions and matched healthy control participants (*n* = 17). Results are shown from 2 (group: lorazepam/placebo or lesion/control) × 3 (threat: low, medium, or high) × 2 (task: active or passive) × 15 (time bins of 1 second each) analyses of variance. Overall condition effects are presented as *F* values; polynomial contrasts and the overall effects of task and group are presented as signed *t* values. Results are Greenhouse-Geisser corrected for violations of multisphericity, and secondary measures are Bonferroni corrected for six measures per experiment. (For ease of comparison across primary and secondary measures, the table lists *p* values multiplied by the number of measures in the correction. Resulting values exceeding 1 are stated as 1.) Linear contrasts are coded as higher dependent values for lorazepam than placebo or for patients than control participants, for higher levels of threat, and for later time points. For more results on both studies, see Supplemental Figure S1. For more results of the lorazepam study, see also Supplemental Tables S1 to S4. For more results of the amygdala study, see also Supplemental Figure S3 and Supplemental Tables S5 and S6.

a Corrected significance level: *p* < .05.

To address whether the less cautious strategy induced by lorazepam is maladaptive in our ACC task, we compared the average number of tokens retained at the end of each epoch, including the chase phase. No significant overall group difference emerged (*p* > .10) (Supplemental Table S1). This implies that both groups maximize token collection in the game, but by using different strategies.

There was no group difference in explicit ratings of predator probability or preference for the three predators (all *p*s > .10) (Supplemental Table S1), and including these measures as covariates (together with their time interaction) led to the same pattern of results for the individual metrics (Supplemental Table S2) and the loss adaptation score (Supplemental Table S1). Within the placebo group, participants’ behavior was influenced by threat level and was generally similar to the behavior of healthy participants in our previous report (15) (Supplemental Figures S1 and S2 and Supplemental Table S3).

### Sedation Does Not Explain the Effects of Lorazepam on Anxiety-like Behavior

Saccadic peak velocity, a sensitive measure of benzodiazepine-induced drowsiness (40, 46, 47, 48, 49, 50), did not differ between groups immediately before and after the game (*p* > .10) (see Supplemental Methods and Supplemental Table S4). Including saccadic peak velocity (measured pre- or posttask) as a covariate (together with its time interaction) did not change the results of the group comparison for any of our outcome measures (Supplemental Tables S2 and S4). Furthermore, reaction times in the game (i.e., escape latencies [when the predator woke up]) did not differ between the groups (*p* > .10) (Supplemental Table S1).

### Two Patients With Congenital Amygdala Lesions Show Reduced Anxiety-like Behavior Relative to Healthy Controls

Patients with amygdala lesions, compared with the control group, spent less time in the safe quadrant as intra-epoch time progressed (*t* = −4.5, *p* = .0001) (Table 1 and Figure 2). A similar pattern was found in five of the six secondary measures after Bonferroni correction (Table 1 and Figure 3); compared with control participants, patients with lesions kept relatively less distance from the threat (*t* = −5.2, *p* = .0002) and more distance from the nearest wall (*t* = 4.1, *p* = .0013); they were less often in the safe place (*t* = −6.2, *p* < .0001), collected more tokens (*t* = 4.7, *p* = .0002), and had higher speed when outside the safe place (*t* = 3.7, *p* = .0031) as the epoch progressed. Again, this was reflected in a smaller loss adaptation score (*t*_17_ = 1.7, *p* = .0539) (Figure 4).

The overall number of tokens retained at the end of all epochs did not differ between groups (*p* > .10) (Supplemental Table S5). There was no evidence for general motor slowing in the lesion group given that escape latencies were comparable between groups (*p* > .10) (Supplemental Table S5).

The two groups showed trendwise group × threat interactions in two measures of explicit threat knowledge, such that the patients with amygdala lesions estimated the wake-up of the predator with the highest threat level as particularly probable (*t* = 1.8, *p* = .0763) and early (*t* = −1.8, *p* = .0892) (Supplemental Table S5). However, no overall group difference emerged (all *p*s > .10), and a covariance analysis revealed that the difference in loss adaptation score was in a similar range when accounting for these subjective reports (Supplemental Table S5). Overall behavior of the control group was comparable to our previous results in healthy participants (15) (Supplemental Figures S1 and S2 and Supplemental Table S6).

### Amygdala and Hippocampus Lesions Had Comparable Behavioral Effects

Finally, we quantitatively compared the effects due to lesions to the amygdala, as demonstrated in the current report, and to hippocampus lesions, as shown in our previous report (15). Of note, lesions were selective to the amygdala or the hippocampus (for detailed descriptions, see Methods and Materials). The calcifications in the two Urbach-Wiethe patients did not extend to the hippocampus itself, and the patients previously scored normal on memory tests (including a German version of the Rey Auditory Verbal Learning Test). Conversely, amygdala tissue was spared in the patients with hippocampus lesions.

We used a 2 (lesion/control) × 2 (study group: amygdala/hippocampus) ANOVA (see also Figure 4). In this analysis, dissociation between the effect of hippocampus and amygdala lesions would be revealed as a lesion × study group × time interaction, and an overall impact of lesions would be revealed as a lesion × time interaction. We found a lesion × time interaction for the primary measure, presence in the safe quadrant (*t* = −9.6, *p* < .0001), and for five out of six secondary measures (distance from threat: *t* = −10.0, *p* < .0001; distance from nearest wall: *t* = −9.0, *p* < .0001; presence in safe place: *t* = −6.5, *p* < .0001; presence in threat quadrant: *t* = 5.0, *p* < .0001; token collection: *t* = 8.5, *p* < .0001) (Figures 2 and 3). A significant lesion × study group × time interaction was found for one of the metrics (presence in safe place: *t* = −3.7, *p* = .0017). However, post hoc comparisons showed that in this measure the two control groups differed more than the lesion groups, and the descriptive pattern suggests that floor effects contributed to this interaction (Figure 3; see also Supplemental Figure S2). Relatedly, a main effect of lesion emerged on the loss adaptation score (*F*_1,34_ = 8.1, *p* = .0074), but there was no significant lesion × study group interaction and no main effect of study group (both *p*s > .10).

## Discussion

Anxiolytics influence both rodent AAC behavior and human clinical anxiety, and this is often taken to suggest that AAC is a valid preclinical anxiety model (51). Here, we demonstrated that the benzodiazepine lorazepam reduces anxiety-like behavior in human AAC. We then investigated 2 patients with selective bilateral amygdala lesions due to congenital Urbach-Wiethe disease and showed that these patients exhibited reduced anxiety-like behavior. Interestingly, the behavior of these 2 patients with amygdala lesions does not differ from a previously investigated group of individuals with hippocampal sclerosis.

Detailed control analyses make it unlikely that drug side effects are responsible for the impact of lorazepam. Reaction times, both in the beginning of an epoch and when the virtual predator became active, did not differ between groups. Furthermore, we did not observe group differences in saccadic eye movements, a sensitive marker of benzodiazepine-induced sedation, both immediately before and after the AAC test. Overall, our results suggest that behavioral effects of benzodiazepines in human AAC are homologous to those in classic rodent AAC. This provides a neuropharmacological validation of our virtual AAC paradigm and thus distinguishes it from more common human anxiety tests such as public speaking anticipation, which is not sensitive to anxiolytic drugs and instead responds to acute administration of serotonergic substances (52).

Benzodiazepines bind allosterically to GABA_A_ receptors and thereby increase the inhibitory action of GABA (28). Among other regions, GABA_A_ receptors are prominently expressed in the amygdala in both rodents (27, 53) and humans (54), which points to a possible role of the amygdala in anxiety-related behaviors. In addition, electrophysiological recordings in rodents during an AAC test have recently indicated a role of the amygdala (26) in addition to previous demonstrations of hippocampal involvement (55). Functional neuroimaging studies in humans suggest that the amygdala may mediate the anxiolytic effect of lorazepam in an emotional face assessment task (31). By demonstrating a critical role of amygdala lesions in human AAC, our findings offer a missing link between assessments of human and rodent anxiety. Strikingly, we observed similar effects as in the lorazepam group: as intra-epoch time passed, patients with lesions spent less time in the safe quadrant and safe place, collected more tokens, and were faster outside the safe place compared with a control group.

These findings in the group with amygdala lesions qualitatively, and indeed quantitatively, mirror the reduction in loss adaptation that we have previously reported for a group with hippocampus lesions (15) (see Figures 2 and 3). This raises the pertinent question of whether amygdala and hippocampus lesions affect the same or complementary components. Potentially, the two structures could mediate approach versus avoidance behaviors in a differential manner that is not immediately obvious in our task. Our measures of anxiety-like behavior can result from decreased approach and/or increased avoidance and may distinctly relate to influences of the hippocampus and the amygdala, respectively. Paradigms with better segregation of individual actions are required to answer this question (17).

Our results relate to two recent studies of nonhuman primates making cost–benefit decisions between unpleasant stimuli and liquid rewards (56, 57). In one of these two studies, benzodiazepine administration increased approach decisions during AAC (56). In addition, benzodiazepine partly reversed the effects of electric stimulation to the pregenual cingulate cortex, which shifted the baseline approach–avoidance balance toward avoidance (56). In another monkey study, disrupting the amygdala-hippocampus circuit abolished an approach–avoidance imbalance that was induced by inactivating the anterior orbitofrontal cortex (57). In human anxiety-like behavior, the interplay of cingulate and orbitofrontal cortex with amygdala-hippocampus circuits remains to be investigated.

The bed nucleus of the stria terminalis (BNST) represents an additional candidate region that can be expected to influence anxiety-like behavior because it is interconnected with the amygdala as well as the hippocampus (58, 59). An abundance of rodent studies (32, 60) and a burgeoning literature in humans has implicated the BNST (58, 61, 62) in sustained responses to uncertain threats. For example, the BNST was related to fear-potentiated startle responses (32, 61), where electric shocks are either cued or unpredictable, or to direct exposure to phobia-eliciting stimuli such as spiders (63, 64). Although there is no evidence of BNST lesions in our sample, the amygdala provides important BNST inputs that are likely to be damaged in our patients with lesions.

Hippocampal sclerosis and Urbach-Wiethe disease, the two lesion models investigated in this study and our previous report (15), gradually encroach during brain development. It is possible that other brain structures can over time compensate for some loss of function due to such deteriorating hippocampus or amygdala deficiencies. Thus, these lesion models are not directly comparable to acute brain lesions in nonhuman animals, and we cannot disentangle whether acute or developmental effects underlie the observed impact of lesions on anxiety behavior. Testing patients with acute lesions could mitigate such concerns. In addition, we cannot dissect the precise subregions of the amygdala or the hippocampus responsible for the observed effects, and we cannot entirely rule out an impact of damage to traversing or nearby fiber tracts.

Interestingly, some drugs that affect the GABAergic system, such as valproic acid, appear to be anxiolytic in rodent AAC but have not been validated in clinical conditions (65, 66, 67). The reason for this discrepancy is as yet unclear. With our AAC paradigm, we furnish a platform for preclinical drug testing in humans, thereby elucidating possible species differences in anxiolytic drug action.

In conclusion, we investigated the impact of benzodiazepines and amygdala lesions on behavior in a human analogue of animal AAC paradigms, which are extensively used in the preclinical evaluation of anxiolytics (1, 2, 3). We demonstrated that loss adaptation in our paradigm, a critical measure of anxiety-like behavior, is reduced both by benzodiazepines and by amygdala lesions. Furthermore, there is no qualitative difference, and indeed no appreciable quantitative difference, between the effects of amygdala lesions and hippocampus lesions on anxiety-like behavior. This provides a crucial link between investigations on animal models of anxiety, which have often focused on the rodent hippocampus (20, 21), and research on human anxiety, which tends to stress the role of the amygdala (30, 68, 69, 70, 71). In a wider context, our approach of using behavioral measures in a well-defined paradigm is in line with a recent proposal that emphasizes the need to dissociate behavioral symptoms and subjective experience of anxiety in basic research (72, 73) and in clinical conditions (51). By suggesting a missing link between human and animal work on anxiety, we hope to have advanced the understanding of the neural mechanisms supporting anxiety-like behavior.

## Acknowledgments and Disclosures

This work was funded by the University of Zurich (to DRB). The Wellcome Trust Centre for Neuroimaging is supported by a strategic grant from the Wellcome Trust (Grant No. 091593/Z/10/Z).

DRB, JV, and CWK designed the study. JV, RH, LF, JM, and DRB conducted the research. DRB and CWK analyzed the data. All authors contributed to the final manuscript.

We thank Matthias Staib, Jennifer Hueber, and Dirk Scheele for help with data acquisition.

All authors report no biomedical financial interests or potential conflicts of interest.

## ARTICLE INFORMATION

From the Division of Clinical Psychiatry Research (CWK, JV, DRB), Psychiatric Hospital, and Neuroscience Center Zurich (CWK, JV, DRB), University of Zurich, Zurich, Switzerland; Epilepsy Unit (JM), University Hospital of Bellvitge, Cognition and Brain Plasticity Unit (LF), Institute of Biomedicine Research of Bellvitge, Department of Cognition, Development, and Educational Psychology (LF), and Institute of Neurosciences (LF), University of Barcelona, Barcelona, Spain; Department of Psychiatry and Division of Medical Psychology (RH), University of Bonn, Bonn, Germany; and Wellcome Trust Centre for Neuroimaging (DRB), University College London, London, United Kingdom

Address correspondence to Christoph W. Korn, Ph.D., Institute for Systems Neuroscience, University Medical Center Hamburg-Eppendorf, Martinistrasse 52, 20246 Hamburg, Germany; E-mail: c.korn@uke.de.

Received Oct 12, 2016; revised Jan 18, 2017; accepted Feb 1, 2017.
