# Supplementary material for: Amygdala Lesions Reduce Anxiety-like Behavior in a Human Benzodiazepine-Sensitive Approach–Avoidance Conflict Test
Source: Biol Psychiatry. 2017 Oct 1;82(7):522–31. doi: 10.1016/j.biopsych.2017.01.018 (PMC5598543; doi:10.1016/j.biopsych.2017.01.018)
Supplement: Supplementary file 1 — Supplementary material [file mmc1.pdf]

Amygdala Lesions Reduce Anxiety-Like Behavior in a Human Benzodiazepine-Sensitive Approach-Avoidance Conflict Test

Supplementary Information

Supplementary Data Items

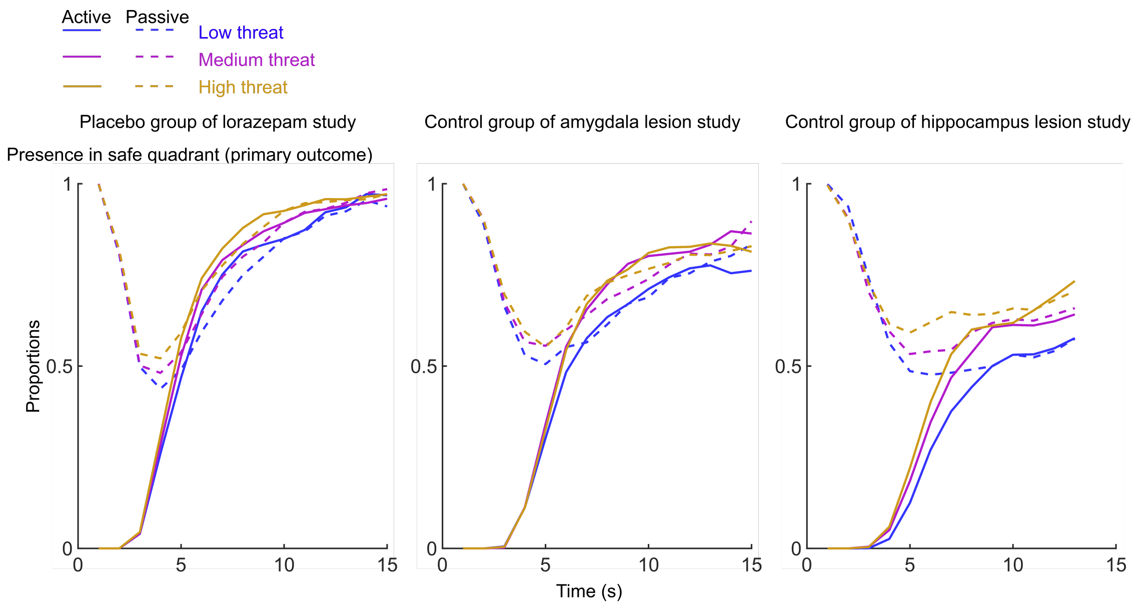

Figure S1. Overall behavior of the control groups in the primary outcome measure of loss adaptation with respect to threat level (related to **Table 1** and **Figure 2**). See also **Figure S2** and **Tables S3 & S6**.

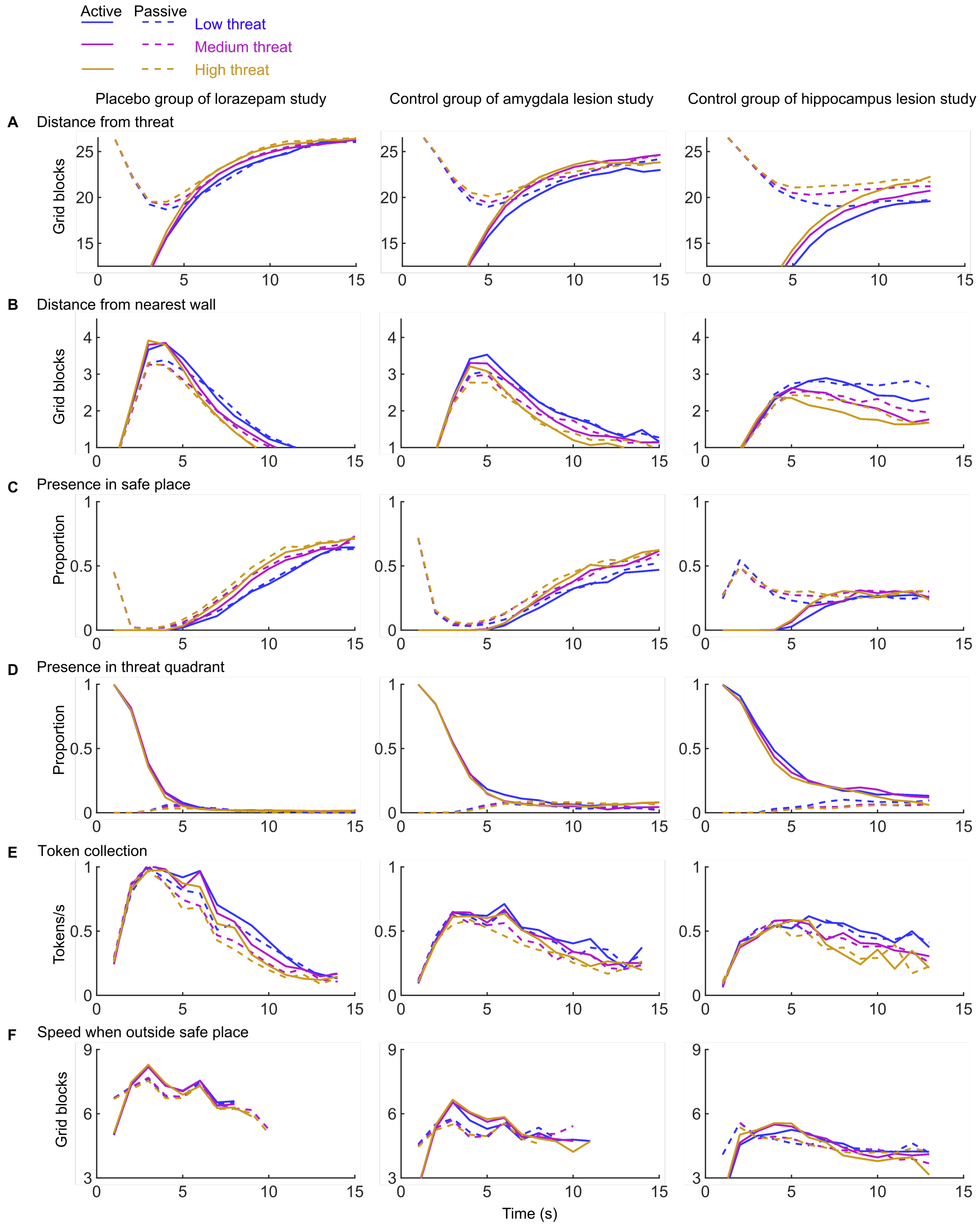

Figure S2. Overall behavior of the control groups in the six secondary outcome measures loss adaptation with respect to threat level (related to **Table 1** and **Figure 3**). See also **Figure S1** and **Tables S3 & S6**. The general pattern was comparable across all three control groups. The beginning of the time course of “presence in the safe place” is slightly different between the previous hippocampus lesion study and the present experiments. In the previous study, participants moved out of the safe place if they had pressed the cursor key during preview phase preceding each epoch. This feature enabled unintentional moves and participants often went back to the safe place shortly afterwards. In the present experiments, key presses were only allowed after the preview. Importantly, this detail had no bearing on behavior as intra-epoch time progressed.

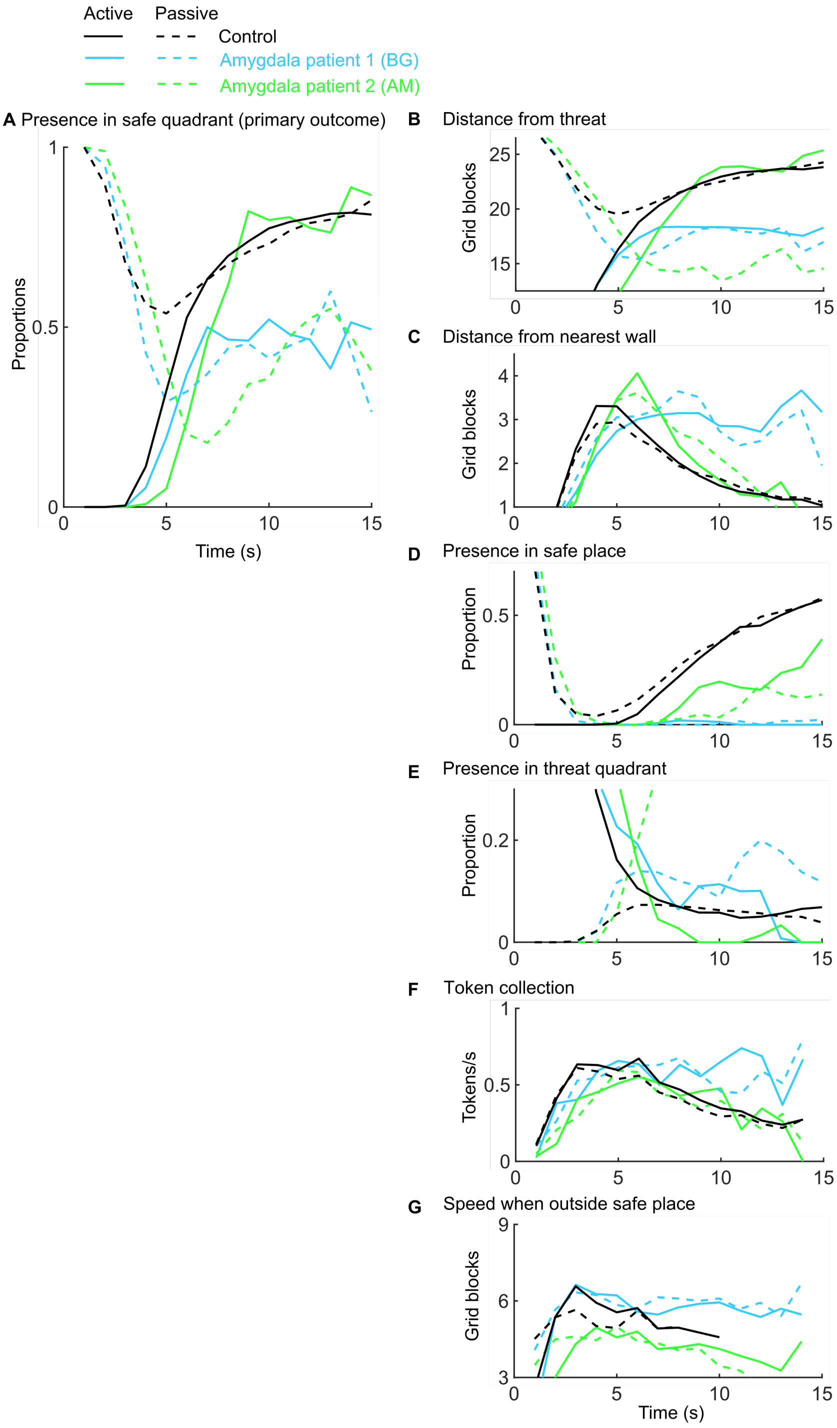

Figure S3. Data from the two patients with amygdala lesions plotted separately (see **Figures 2 & 3**). In previous reports, patient 1 and patient 2 were labelled BG and AM, respectively (1–3). When analyzing the two patients together, significant group × time interactions were observed in the primary outcome, presence in the safe quadrant (**A**) as well as in the following secondary outcomes: Presence in the safe place (**D**), token collection (**F**), and speed outside the safe place (**G**). Trends emerged for distance from threat (**B**) and from the nearest wall (**C**). The graphs reveal qualitative differences of both patients in most of these measures (with the exception of token collection and speed outside the safe place). In most measures (expect for speed outside the safe place) patient 1 seemed to show greater qualitative differences more from the control group than patient 2. This was also reflected in a lower loss adaptation score for patient 1 compared with patient 2 (0.04 versus 0.14; mean ± SD of the

control group 0.28 ± 0.15; see **Figure 4**). The somewhat greater differences for patient 1 (BG) versus patient 2 (AM) are in line with previous reports also reporting greater differences for patient 1: Patient 1 showed different empathy ratings on both positive and negative items but patient 2 only exhibited different empathy ratings for positive items (4). A study by Becker et al. 2012 (5) showed that patient 1 was impaired in recognizing fearful faces, exhibited aberrant startle responses, and had an unusually small social network. In all of these measures, patient 2 was similar to controls (5).

Table S1. Lorazepam versus placebo groups. Additional intra-epoch-time-independent measures (related to **Table 1**).

	During the experiment								Post-experiment					
Measure/Effect	Tokens collected		Foraging latency		Escape latency		Tokens retained		Subjective wake-up probability		Subjective wake-up time		Choice for last round	
	F-/t-values	p-values	F-/t-values	p-values	F-/t-values	p-values	F-/t-values	p-values	F-/t-values	p-values	F-/t-values	p-values	F-/t-values	p-values
Group	-0.84	.4065	*-1.76*	*.0830*	0.12	.9049	-1.36	.1804	0.60	.5538	-0.09	.9324	1.37	.1751
Group × Threat overall	0.24	.7894	1.40	.2517	1.35	.2637	0.84	.4351	0.34	.7153	0.76	.4694	0.06	.9453
Group × Threat linear	-0.13	.8950	-1.39	.1658	-1.20	.2335	-1.27	.2079	-0.80	.4255	-0.28	.7777	0.34	.7379
Group × Task	**2.38**	**.0207**	-0.55	.5827	0.58	.5639	**2.03**	**.0471**	NA	NA	NA	NA	NA	NA
Group × Threat × Task overall	0.04	.9604	**3.17**	**.0456**	0.03	.9689	0.11	.8988	NA	NA	NA	NA	NA	NA
Group × Threat × Task linear	0.07	.9470	1.36	.1758	0.23	.8219	0.40	.6903	NA	NA	NA	NA	NA	NA

Comparison of lorazepam-treated (n=29) and placebo-treated (n=30) participants. Results are shown from a 2 (Group: Lesion/Control) × 3 (Threat: Low, Medium, High) × 2 (Task: Active/Passive) ANOVA. Overall condition effects are presented as F-values; polynomial contrasts and the overall effects of task and group are presented as signed t-values. Linear contrasts are coded as higher dependent values for lorazepam than placebo and with higher levels of threat. Significance levels: p<.1 in italics; p<.05 in bold.

Before predator wakeup, participants under lorazepam compared to placebo collected relatively fewer tokens in the passive condition (group × task interaction). In the active starting position, participants under lorazepam started foraging earlier than those under placebo in the medium and high threat levels (group × threat × task interaction).

The same pattern of results for the group comparison of loss adaptation scores as reported in the main text was obtained when including the post-experiment variables as covariates: subjective wake-up probability: F(1,56)=5.1; p=.0141; subjective wake-up time: F(1,56)=5.1; p=.0136; chosen predator type in bonus round: F(1,56)=5.3; p.0128.

Table S2. Lorazepam versus placebo groups. Seven metrics of loss adaptation: Covariate analyses for the effect of “Group × Time linear” (related to **Table 1**).

Covariate	Presence in safe quadrant		Distance from threat		Distance from nearest wall		Presence in safe place		Presence in threat quadrant		Token collection		Speed when outside safe place	
	F-/t-values	p-values	F-/t-values	p-values	F-/t-values	p-values	F-/t-values	p-values	F-/t-values	p-values	F-/t-values	p-values	F-/t-values	p-values
Subjective wake-up probability	**-4.18**	**.0090**	*-4.05*	*.0688*	**5.28**	**.0214**	-5.38	.0307	0.07	1	**5.22**	**.0174**	-1.07	1
Subjective wake-up time	**-4.31**	**.0078**	*-4.16*	*.0623*	**5.15**	**.0245**	-5.32	.0322	-0.01	1	**5.07**	**.0208**	1.47	.7964
Choice for last round	**-4.45**	**.0067**	*-4.33*	*.0525*	**5.29**	**.0212**	-5.38	.0306	-0.06	1	**5.35**	**.0151**	1.47	.7970
Saccadic peak velocity: pre-task	**-4.04**	**.0105**	*-3.94*	*.0774*	**5.15**	**.0246**	-5.45	.0290	-0.23	1	**5.38**	**.0144**	1.48	.7851
Saccadic peak velocity: post-task	**-4.14**	**.0094**	*-4.01*	*.0720*	**5.07**	**.0267**	*-5.30*	*.0327*	-0.02	1	**5.23**	**.0174**	1.39	.8623

Including the listed covariates resulted in similar effects of “Group × Time linear” as reported in **Table 1** (i.e., without covariates). Results are Greenhouse-Geisser corrected for violations of multisphericity, and Bonferroni-corrected for secondary outcomes. (For ease of comparison across primary and secondary measures, the table lists p-values multiplied by the number of measures in the correction. Resulting values exceeding 1 are stated as 1.) Linear contrasts are coded as higher dependent values for higher levels of threat, and later time points. Significance levels: $p<.1$ in italics; $p<.05$ in bold.

Table S3. Placebo group of lorazepam study. Overall behavior in the seven metrics of loss adaptation (related to **Table 1**).

Measure/Effect	Presence in safe quadrant		Distance from threat		Distance from nearest wall		Presence in safe place		Presence in threat quadrant		Token collection		Speed when outside safe place	
	F-/t-values	p-values	F-/t-values	p-values	F-/t-values	p-values	F-/t-values	p-values	F-/t-values	p-values	F-/t-values	p-values	F-/t-values	p-values
Threat overall	**9.52**	**.0038**	**8.19**	**.0104**	**9.98**	**.0033**	**9.83**	**.0037**	1.48	1	**9.56**	**.0049**	3.26	.4231
Threat linear	**4.36**	**<.0001**	**4.05**	**.0009**	**-4.40**	**.0003**	**4.39**	**.0003**	-1.25	1	**-4.37**	**.0003**	*-2.55*	*.0814*
Threat quadratic	-0.15	-1	-0.09	1	0.78	1	-0.62	1	-1.18	1	0.26	1	-0.19	1
Task	**26.38**	**<.0001**	**31.32**	**<.0001**	**-3.26**	**.0197**	**6.61**	**<.0001**	**-37.27**	**<.0001**	**-7.89**	**<.0001**	-0.30	1
Time overall	**519.04**	**<.0001**	**919.77**	**<.0001**	**284.28**	**<.0001**	**143.87**	**<.0001**	**1550.31**	**<.0001**	**250.52**	**<.0001**	**76.94**	**<.0001**
Threat × task overall	1.03	1	1.97	1	1.64	1	0.79	1	1.00	1	1.74	1	0.40	1
Task × time overall	**747.04**	**<.0001**	**1471.95**	**<.0001**	**14.17**	**<.0001**	**56.82**	**<.0001**	**1465.49**	**<.0001**	**13.85**	**<.0001**	**70.97**	**<.0001**
Threat × time overall	**3.83**	**.0035**	**4.60**	**.0015**	**4.62**	**.0002**	**5.38**	**.0001**	**3.13**	**.0210**	**4.43**	**<.0001**	1.26	1
Threat × time linear	-0.70	1	0.51	1	**-3.99**	**.0005**	**7.44**	**<.0001**	**4.11**	**.0003**	**-3.96**	**.0005**	**-3.71**	**.0017**
Threat × time quadratic	-0.60	1	0.17	1	0.66	1	-1.12	1	0.26	1	0.96	1	-0.17	1
Threat × task × time overall	0.73	1	0.51	1	0.80	1	0.46	1	1.25	1	1.51	.5147	1.05	1

Placebo-treated participants (n=30). Results are shown from a 3 (Threat: Low, Medium, High) × 2 (Task: Active/Passive) × 15 (Time bins of 1 s each) ANOVA. Overall condition effects are presented as F-values; polynomial contrasts and the overall effect of task are presented as signed t-values. Results are Greenhouse-Geisser corrected for violations of multisphericity, and Bonferroni-corrected for all metrics. (For ease of comparison across primary and secondary measures, the table lists p-values multiplied by the number of measures in the correction. Resulting values exceeding 1 are stated as 1.) Linear contrasts are coded as higher dependent values for higher levels of threat, and later time points. Significance levels: p<.1 in italics; p<.05 in bold. See also **Figure S1**.

Table S4. Lorazepam versus placebo groups. Saccadic eye movements as control measure for sedative effects (related to **Table 1**).

Measure/Effect	Saccadic peak velocity		Saccadic latency	
	F-values	p-values	F-values	p-values
Group	F(1,57)=0.16	.6853	F(1,57)=0.79	.3780
Time	F(1,57)=0.22	.6393	F(1,57)=0.27	.6059
Group × Time	F(1,57)=0.01	.9448	F(1,57)=0.48	.8273

Results are shown from a 2 (Group: Lorazepam/Placebo) × 2 (Time: Before/After task) ANOVA. None of the effects reached significance. Including saccadic peak velocity (measured pre- or post-task) as a covariate did not change the results of the group comparison for the loss adaptation score (pre-task: F(1,56)=5.0; p=.0294; post-task: F(1,56)=4.9; p=.0304).

Table S5. Amygdala lesions versus healthy controls. Additional intra-epoch-time-independent measures (related to **Table 1)**

Measure/Effect	During the experiment								Post-experiment					
	Tokens collected		Foraging latency		Escape latency		Tokens retained		Subjective wake-up probability		Subjective wake-up time		Choice for last round	
	F-/t-values	p-values	F-/t-values	p-values	F-/t-values	p-values	F-/t-values	p-values	F-/t-values	p-values	F-/t-values	p-values	F-/t-values	p-values
Group	-0.29	.7746	87.08	**<.0001**	0.23	.8587	-0.87	.3958	-1.33	.1996	-0.67	.5089	-1.22	.2373
Group × Threat overall	0.04	.9576	1.80	.1803	0.09	.9235	0.84	.4389	**3.48**	**.0422**	1.92	.1622	0.12	.8834
Group × Threat linear	0.09	.9326	1.23	.2268	-0.11	.9299	-1.12	.2706	*1.83*	*.0763*	*-1.75*	*.0892*	0.49	.6274
Group × Task	**2.61**	**.0184**	**2.36**	**.0306**	0.13	.9181	0.45	.6562	NA	NA	NA	NA	NA	NA
Group × Threat × Task overall	1.71	.1953	**10.46**	**.0003**	1.38	.2661	0.20	.8197	NA	NA	NA	NA	NA	NA
Group × Threat × Task linear	*1.79*	*.0819*	0.65	.5205	1.3	.2039	-0.34	.7361	NA	NA	NA	NA	NA	NA

Comparison of two patients with bilateral amygdala lesions and matched healthy controls (n=17). Results are shown from a 2 (Group: Lesion/Control) × 3 (Threat: Low, Medium, High) × 2 (Task: Active/Passive) ANOVA. Overall condition effects are presented as F-values; polynomial contrasts and the overall effects of task and group are presented as signed t-values. Linear contrasts are coded as higher dependent values for patients than controls and with higher levels of threat. Significance levels: p<.1 in italics; p<.05 in bold.

The number of tokens collected before predator wakeup showed a significant group × task interaction such that the amygdala group collected relatively less tokens in the passive condition. Foraging latencies showed a significant triple interaction of group × threat × task overall similar as in the lorazepam study.

A similar pattern of results for the group comparison of loss adaptation scores as reported in the main text was obtained when including the post-experiment variables as covariates: subjective wake-up probability: $F(1,16)=1.9$; $p=.0902$; subjective wake-up time: $F(1,16)=3.2$; $p=.04705$; chosen predator type in bonus round: $F(1,16)=2.6$; $p=.0649$.

Table S6. Control group of amygdala lesions study. Overall behavior in the seven metrics of loss adaptation (related to **Table 1**).

Measure/Effect	Presence in safe quadrant		Distance from threat		Distance from nearest wall		Presence in safe place		Presence in threat quadrant		Token collection		Speed when outside safe place	
	F-/t-values	p-values	F-/t-values	p-values	F-/t-values	p-values	F-/t-values	p-values	F-/t-values	p-values	F-/t-values	p-values	F-/t-values	p-values
Threat overall	5.16	.1223	3.83	.2972	*6.30*	*.0978*	*6.03*	*.0942*	0.65	1	**8.17**	**.0310**	0.10	1
Threat linear	*2.85*	**.0076**	2.54	*.0967*	**-3.55**	**.0073**	**3.40**	**.0109**	0.31	1	**-4.01**	**.0020**	-0.46	1
Threat quadratic	-1.48	1	-1.09	1	-0.02	1	-0.70	1	1.10	1	0.51	1	0.03	1
Task	**15.38**	**<.0001**	**18.61**	**<.0001**	-1.08	1	**6.58**	**<.0001**	**-23.35**	**<.0001**	**-5.03**	**.0009**	-0.95	1
Time overall	**45.42**	**<.0001**	**78.94**	**<.0001**	**39.21**	**<.0001**	**30.44**	**<.0001**	**182.16**	**<.0001**	**34.08**	**<.0001**	**79.20**	**<.0001**
Threat × task overall	0.75	1	2.83	.5148	0.62	1	0.21	1	4.18	.171	1.38	1	**7.58**	**.0142**
Task × time overall	**265.41**	**<.0001**	**502.40**	**<.0001**	**4.90**	**.0037**	**77.24**	**<.0001**	**307.61**	**<.0001**	2.15	.1525	**68.50**	**<.0001**
Threat × time overall	1.87	.6343	2.81	.1084	2.08	.3427	2.44	.3800	2.19	.1340	1.75	.3414	**2.75**	**.0164**
Threat × time linear	2.01	**.0474**	0.23	1	*-3.95*	**.0008**	**7.01**	**<.0001**	**4.61**	**<.0001**	*-3.01*	**.0175**	-1.34	1
Threat × time quadratic	-3.03	.1890	**-4.37**	**.0452**	-0.30	1	-1.55	.6753	2.68	.209	1.27	1	-0.74	1
Threat × task × time overall	1.19	1	1.86	.4694	0.62	1	0.69	1	1.20	1	1.6	.4658	0.64	1

Healthy controls (n=17). Results are shown from a 3 (Threat: Low, Medium, High) × 2 (Task: Active/Passive) × 15 (Time bins of 1 s each) ANOVA. Overall condition effects are presented as F-values; polynomial contrasts and the overall effect of task are presented as signed t-values. Results are Greenhouse-Geisser corrected for violations of multisphericity, and Bonferroni-corrected for all metrics. (For ease of comparison across primary and secondary measures, the table lists p-values multiplied by the number of measures in the correction. Resulting values exceeding 1 are stated as 1.) Linear contrasts are coded as higher dependent values for higher levels of threat, and later time points. Significance levels: p<.1 in italics; p<.05 in bold. See also **Figure S1**.

Supplementary Methods

Lorazepam study: In-, and exclusion criteria, medical screening, and study registration

Healthy, German-speaking participants between 18 and 40 years of age were included. One female participant from the lorazepam group was excluded post-hoc because of elevated TSH levels, which indicated a possible thyroid condition. All participants had normal or corrected-to-normal vision; no participant wore glasses.

Exclusion criteria were: 1) contraindications to benzodiazepines (dependence, history of allergic reactions, history of hypotonia associated with benzodiazepines), 2) drug use in the two weeks prior to the study (with the exception of contraceptive drugs and incidental use of NSARs or paracetamol), 3) known or suspected drug or alcohol abuse, 4) any history of psychiatric, neurological, or systemic/rheumatic disease, 5) other clinically significant concomitant disease states (e.g., renal failure, hepatic dysfunction, cardiovascular disease, thyroid condition, etc.), 6) pregnant or breast-feeding women, women intending to become pregnant, or women who did not use a safe method of contraception, 7) participation in another drug study within the 30 days preceding and during the present study.

After a short pre-screening via a telephone interview, potentially eligible participants were invited for a medical examination, maximally one week prior to testing. An independent physician assessed participants' medical history and performed a physical examination, to check exclusion criteria. Participants gave blood and urine samples which were screened for signs of hematological/metabolic, hepatic, renal, and thyroid disease, blood cell count, electrolytes, C-reactive protein, aspartate aminotransferase (ASAT/GOT), alanine aminotransferase (ALAT/GPT), gamma-glutamyl transferase (gamma-GT), kreatinin, thyroid-stimulating hormone (TSH), free thyroxine (FT4). Urine samples were further screened for previous drug use (amphetamines, barbiturates, benzodiazepines, tetrahydrocannabinol, cocaine, methadone, and opioids). Female participants were additionally screened for pregnancy using a urine beta human chorionic gonadotropin (beta-HCG) test.

Lorazepam study: Details on study medication

The study dose of 1 mg was based on the smallest effective dose, as recommended by the manufacturer, in order to reduce side effects. The Kantonsapotheke Zürich (Pharmacy of the Canton of Zurich) manufactured, blinded and randomized the study medication: 30 capsules for men and 30 for women. Each gender lot was randomized into 15 placebo (mannitol), and 15 active drug (lorazepam) capsules. Study enrollment was conducted by J.V.

Lorazepam study: Questionnaires

Before drug administration, participants completed the following questionnaires: a brief current health questionnaire (to check for possible occurrences of new medical conditions), the State Trait Anxiety Inventory (STAI) (6), the Behavioral Avoidance/Inhibition Scales (BIS-BAS) (7), and the Beck Depression Inventory (BDI) (8). The two groups did not differ in any of the acquired measures (all p's>.1).

Lorazepam study: Saccadic eye movements as control measure for sedative effects of lorazepam

To control for the possible sedative effects of lorazepam (9–13), we measured saccadic eye movements as described previously (14). Measurements were obtained before (i.e., 100 min after drug administration) and after the behavioral AAC paradigm. Eye movements of both eyes were recorded using the EyeLink 1000 System (SR Research, Ottawa, Ontario, Canada) at a sampling rate of 500 Hz. Testing was performed in a dark, soundproof chamber. (In contrast, behavioral testing was performed on a different PC in a room with ambient light.) Participants were seated 57 cm from a monitor (Dell P2012H, 60 Hz refresh rate) with their head position fixed onto a chin rest. Calibration was performed using the 9-point calibration procedure implemented in the EyeLink software. The MATLAB toolbox Psychtoolbox (http://psychtoolbox.org/) was used for task presentation. Participants were asked to follow a target with their eyes (i.e., a black dot with a diameter of 0.3° visual angle on a white background). A trial started with the black dot in the center of the screen (0° visual angle). After a random duration of either 1000 or 2000 ms, the dot jumped to one of four

possible peripheral positions (±7.25°, ±14.5°, i.e., left or right of the initial dot position) and remained there for 1000 ms. In total there were 60 trials, i.e., 15 for each position.

The task was analyzed using custom scripts in MATLAB following previously described routines (14). Only saccades with a minimum amplitude of 1° and a minimum onset latency of 100 ms were included. If a saccade or an eye blink occurred between 100 ms before and after dot movement, trials were excluded. We calculated two variables for the first directionally correct saccade in each epoch: 1) saccadic peak velocity (maximum value of the movement derivative in degree per second) and 2) saccadic latency (time in ms from target appearance to saccade initiation). Saccadic peak velocity is considered the most reliable metric for drug-induced drowsiness (9). The two measures were submitted to 2 × 2 mixed-measures ANOVAs [one between-subjects factor: drug (Lorazepam/Placebo) and one within-subjects factor: time point (before/after behavioral AAC)]. None of the metrics revealed a main effect of drug or an interaction including the factor drug. Results are presented in **Table S4**. Additionally, we explored whether there were effects of drug on the two measures when treating saccades to the two peripheral eccentricities separately (i.e., ±7.25°and ±14.5°). No significant effects emerged.

Behavioral approach-avoidance paradigm—additional details

Movements on the grid: Participants coordinated their movements by pressing the four computer keyboard arrow keys. No diagonal movements were enabled. Participants could move at a maximum speed of 10 grid blocks per second. All three predators had the same speed of 40 grid blocks per second. Thus, participants could try to escape an active predator by retreating to the safe place but the predator moved four times faster, and thus escape was only possible if participants were close to the safe place.

Epoch duration: Epochs lasted 3 to 15 s (drawn from a uniform distribution with steps of 1.7 s). There was a 3 s countdown with a preview of the grid layout before each epoch, during which the player could not move. This was meant to facilitate orientation with respect to starting place/predator position. After the pre-determined epoch duration, the predator either woke up, or the next epoch started. This means that different from the previous version of the game (15), the average duration of

the epochs was not different between threat levels, which made it possible to analyze time-independent epoch-summary measures.

Post-task questions: Participants rated on a visual analogue scale (ranging from 0% to 100%) the wake-up probability and the wake-up time of the three different predators.

Bonus epoch: Finally, participants were given the choice to select the predator that they would like to face in a final bonus round. The selection process entailed three consecutive pair-wise comparisons between the predators.

Payment: At the end of the game, the average number of tokens from ten randomly selected epochs was transformed into a monetary reimbursement that was added to the constant show-up fee.

The task was presented using the MATLAB toolbox Cogent (www.vislab.ucl.ac.uk).

Background information on the previously published sample with hippocampus lesions

Epileptic patients (n=7) and healthy controls (n=12) were recruited at Bellevitge University Hospital (15). Patients suffered from seizures with typical temporal lobe features that were not controlled with antiepileptic drugs. Two experienced neurologists and one neuroradiologist scrutinized T1-weighted MRI scans (please refer to Figure S2 in (15) for structural images of all patients). No structural abnormalities apart from uni- or bi-lateral hippocampal sclerosis were found. Volumetric analyses based on a high-resolution T1-weighted MRI scans revealed significant differences in bilateral hippocampal volumes (mean ± SEM volume in ml: hippocampus patients: 3.17 ± 0.41; controls: 4.32 ± 0.50; independent samples t-test: $t(12)=4.4$, $p<.001$) but not in amygdala volume (hippocampus patients: 1.88 ± 0.61; controls: 1.68 ± 0.32; $t(12)=1.1$, $p=0.29$) or in parahippocampal and total intracranial volume (please refer to Tables S3 and S4 in (15) for further information on all patients).

Supplementary References

1. Bach DR, Hurlemann R, Dolan RJ (2015): Impaired threat prioritisation after selective bilateral amygdala lesions. *Cortex*. 63: 206–213.
2. Talmi D, Hurlemann R, Patin A, Dolan RJ (2010): Framing effect following bilateral amygdala lesion. *Neuropsychologia*. 48: 1823–1827.
3. Bach DR, Talmi D, Hurlemann R, Patin A, Dolan RJ (2011): Automatic relevance detection in the absence of a functional amygdala. *Neuropsychologia*. 49: 1302–1305.
4. Hurlemann R, Wagner M, Hawellek B, Reich H, Pieperhoff P, Amunts K, *et al.* (2007): Amygdala control of emotion-induced forgetting and remembering: Evidence from Urbach-Wiethe disease. *Neuropsychologia*. 45: 877–884.
5. Becker B, Mihov Y, Scheele D, Kendrick KM, Feinstein JS, Matusch A, *et al.* (2012): Fear processing and social networking in the absence of a functional amygdala. *Biol Psychiatry*. 72: 70–77.
6. Laux L, Glanzmann P, Schaffner P, Spielberger CD (1981): *STAI - State-Trait-Angstinventar. Theoretische Grundlagen und Handanweisung [in German]*. Weinheim, Germany: Beltz Test GmbH.
7. Carver CS, White TL (1994): Behavioral inhibition, behavioral activation, and affective responses to impending reward and punishment: The BIS/BAS Scales. *J Pers Soc Psychol*. 67: 319–333.
8. Hautzinger M, Bailer M, Worall H (1994): *The Beck Depression Inventory (BDI) [in German]*. Bern, Switzerland: Huber.
9. de Visser SJ, van der Post J, Pieters MS, Cohen AF, van Gerven JM (2001): Biomarkers for the effects of antipsychotic drugs in healthy volunteers. *Br J Clin Pharmacol*. 51: 119–32.
10. Perkins AM, Ettinger U, Weaver K, Schmechtig A, Schrantee A, Morrison PD, *et al.* (2013): Advancing the defensive explanation for anxiety disorders: lorazepam effects on human defense are systematically modulated by personality and threat-type. *Transl Psychiatry*. 3: e246.
11. de Haas SL, de Visser SJ, van der Post JP, Schoemaker RC, van Dyck K, Murphy MG, *et al.* (2008): Pharmacodynamic and pharmacokinetic effects of MK-0343, a GABA(A) alpha2,3 subtype selective agonist, compared to lorazepam and placebo in healthy male volunteers. *J Psychopharmacol*. 22: 24–32.
12. Reilly JL, Lencer R, Bishop JR, Keedy S, Sweeney JA (2008): Pharmacological treatment effects on eye movement control. *Brain Cogn*. 68: 415–435.
13. Masson GS, Mestre DR, Martineau F, Soubrouillard C, Brefel C, Rascol O, Blin O (2000): Lorazepam-induced modifications of saccadic and smooth-pursuit eye movements in humans: Attentional and motor factors. *Behav Brain Res*. 108: 169–180.
14. Schmechtig A, Lees J, Perkins A, Altavilla A, Craig KJ, Dawson GR, *et al.* (2013): The effects of ketamine and risperidone on eye movement control in healthy volunteers. *Transl Psychiatry*. 3: e334.
15. Bach DR, Guitart-Masip M, Packard PA, Miró J, Falip M, Fuentemilla L, Dolan RJ (2014): Human hippocampus arbitrates approach-avoidance conflict. *Curr Biol*. 24: 541–7.

CONSORT 2010 Flow Diagram

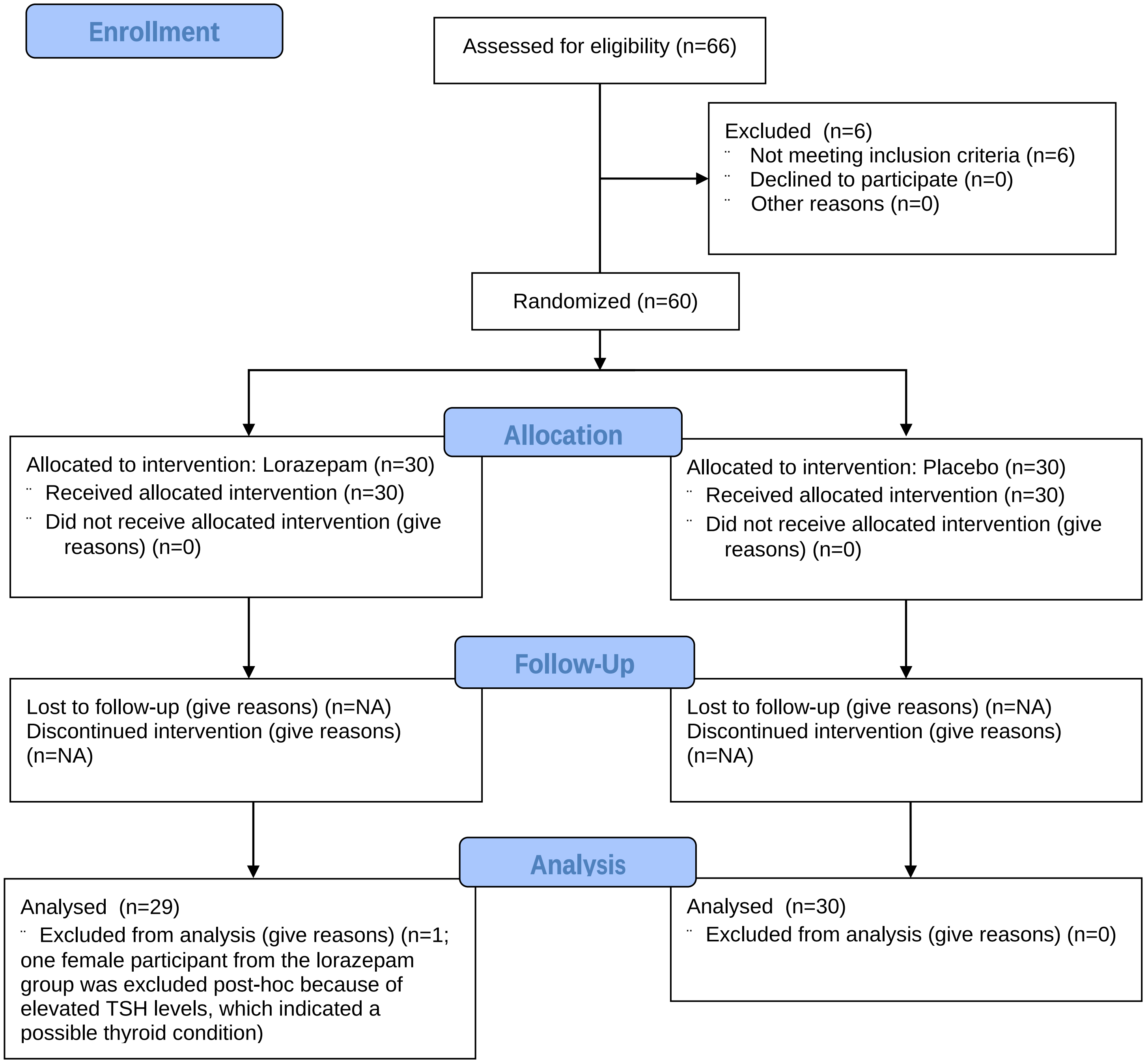

CONSORT 2010 checklist of information to include when reporting a randomised trial*

Section/Topic	Item No	Checklist item	Reported on page No
**Title and abstract**			
	1a	Identification as a randomised trial in the title	NA
	1b	Structured summary of trial design, methods, results, and conclusions (for specific guidance see CONSORT for abstracts)	3
**Introduction**			
Background and objectives	2a	Scientific background and explanation of rationale	4-5
	2b	Specific objectives or hypotheses	5
**Methods**			
Trial design	3a	Description of trial design (such as parallel, factorial) including allocation ratio	6 & SI 17-18
	3b	Important changes to methods after trial commencement (such as eligibility criteria), with reasons	NA
Participants	4a	Eligibility criteria for participants	6 & SI 17
	4b	Settings and locations where the data were collected	6 & SI 17-18
Interventions	5	The interventions for each group with sufficient details to allow replication, including how and when they were actually administered	6 & SI 17-20
Outcomes	6a	Completely defined pre-specified primary and secondary outcome measures, including how and when they were assessed	8-9
	6b	Any changes to trial outcomes after the trial commenced, with reasons	NA
Sample size	7a	How sample size was determined	NA
	7b	When applicable, explanation of any interim analyses and stopping guidelines	NA
Randomisation:			
Sequence generation	8a	Method used to generate the random allocation sequence	SI 17
	8b	Type of randomisation; details of any restriction (such as blocking and block size)	SI 17
Allocation concealment mechanism	9	Mechanism used to implement the random allocation sequence (such as sequentially numbered containers), describing any steps taken to conceal the sequence until interventions were assigned	SI 17
Implementation	10	Who generated the random allocation sequence, who enrolled participants, and who assigned participants to interventions	SI 17
Blinding	11a	If done, who was blinded after assignment to interventions (for example, participants, care providers, those assessing	SI 17

		outcomes) and how	
	11b	If relevant, description of the similarity of interventions	NA
Statistical methods	12a	Statistical methods used to compare groups for primary and secondary outcomes	8-9
	12b	Methods for additional analyses, such as subgroup analyses and adjusted analyses	8-9
**Results**			
Participant flow (a diagram is strongly recommended)	13a	For each group, the numbers of participants who were randomly assigned, received intended treatment, and were analysed for the primary outcome	6 SI 17
	13b	For each group, losses and exclusions after randomisation, together with reasons	6 SI 17
Recruitment	14a	Dates defining the periods of recruitment and follow-up	NA
	14b	Why the trial ended or was stopped	NA
Baseline data	15	A table showing baseline demographic and clinical characteristics for each group	In text 6 SI 17
Numbers analysed	16	For each group, number of participants (denominator) included in each analysis and whether the analysis was by original assigned groups	6, 10
Outcomes and estimation	17a	For each primary and secondary outcome, results for each group, and the estimated effect size and its precision (such as 95% confidence interval)	10, 28, Table 1
	17b	For binary outcomes, presentation of both absolute and relative effect sizes is recommended	NA
Ancillary analyses	18	Results of any other analyses performed, including subgroup analyses and adjusted analyses, distinguishing pre-specified from exploratory	10-11, SI
Harms	19	All important harms or unintended effects in each group (for specific guidance see CONSORT for harms)	NA
**Discussion**			
Limitations	20	Trial limitations, addressing sources of potential bias, imprecision, and, if relevant, multiplicity of analyses	14-15
Generalisability	21	Generalisability (external validity, applicability) of the trial findings	13-15
Interpretation	22	Interpretation consistent with results, balancing benefits and harms, and considering other relevant evidence	13-15
**Other information**			
Registration	23	Registration number and name of trial registry	6
Protocol	24	Where the full trial protocol can be accessed, if available	6
Funding	25	Sources of funding and other support (such as supply of drugs), role of funders	16

*We strongly recommend reading this statement in conjunction with the CONSORT 2010 Explanation and Elaboration for important clarifications on all the items. If relevant, we also recommend reading CONSORT extensions for cluster randomised trials, non-inferiority and equivalence trials, non-pharmacological treatments, herbal interventions, and pragmatic trials. Additional extensions are forthcoming: for those and for up to date references relevant to this checklist, see www.consort-statement.org.
